# Scavenging of Retinoid Cation Radicals by Urate, Trolox, and α-, β-, γ-, and δ-Tocopherols

**DOI:** 10.3390/ijms20112799

**Published:** 2019-06-07

**Authors:** Malgorzata Rozanowska, Ruth Edge, Edward J. Land, Suppiah Navaratnam, Tadeusz Sarna, T. George Truscott

**Affiliations:** 1 Cardiff Institute of Tissue Engineering and Repair, Cardiff University, Wales CF10 3AX, UK; 2 School of Optometry and Vision Sciences, Cardiff University, Cardiff, Wales CF24 4HQ, UK; 3 Dalton Cumbrian Facility, The University of Manchester, Westlakes Science Park, Moor Row, Cumbria CA24 3HA, UK; Ruth.Edge@manchester.ac.uk; 4 Free Radical Research Facility, Science and Technology Facilities Council (STFC) Daresbury Laboratory, Warrington WA4 4AD, UK; E.Land@mighty-micro.co.uk; 5 Biomedical Sciences Research Institute, University of Salford, Manchester M5 4WT, UK; Navaratnam1000@gmail.com; 6 Department of Biophysics, Faculty of Biotechnology, Jagiellonian University, 30-387 Krakow, Poland; Tadeusz.Sarna@uj.edu.pl; 7 School of Chemical and Physical Sciences, Lennard-Jones Building, Keele University, Staffordshire ST5 5BG, UK; T.G.Truscott@keele.ac.uk

**Keywords:** vitamin A, retinol, retinal, retinoic acid, vitamin E, tocopherol, free radicals, antioxidants, skin, retina

## Abstract

Retinoids are present in human tissues exposed to light and under increased risk of oxidative stress, such as the retina and skin. Retinoid cation radicals can be formed as a result of the interaction between retinoids and other radicals or photoexcitation with light. It has been shown that such semi-oxidized retinoids can oxidize certain amino acids and proteins, and that α-tocopherol can scavenge the cation radicals of retinol and retinoic acid. The aim of this study was to determine (i) whether β-, γ-, and δ-tocopherols can also scavenge these radicals, and (ii) whether tocopherols can scavenge the cation radicals of another form of vitamin A—retinal. The retinoid cation radicals were generated by the pulse radiolysis of benzene or aqueous solution in the presence of a selected retinoid under oxidizing conditions, and the kinetics of retinoid cation radical decays were measured in the absence and presence of different tocopherols, Trolox or urate. The bimolecular rate constants are the highest for the scavenging of cation radicals of retinal, (7 to 8) × 10^9^ M^−1^·s^−1^, followed by retinoic acid, (0.03 to 5.6) × 10^9^ M^−1^·s^−1^, and retinol, (0.08 to 1.6) × 10^8^ M^−1^·s^−1^. Delta-tocopherol is the least effective scavenger of semi-oxidized retinol and retinoic acid. The hydrophilic analogue of α-tocopherol, Trolox, is substantially less efficient at scavenging retinoid cation radicals than α-tocopherol and urate, but it is more efficient at scavenging the cation radicals of retinoic acid and retinol than δ-tocopherol. The scavenging rate constants indicate that tocopherols can effectively compete with amino acids and proteins for retinoid cation radicals, thereby protecting these important biomolecules from oxidation. Our results provide another mechanism by which tocopherols can diminish the oxidative damage to the skin and retina and thereby protect from skin photosensitivity and the development and/or progression of changes in blinding retinal diseases such as Stargardt’s disease and age-related macular degeneration (AMD).

## 1. Introduction

Tocopherols and retinoids with vitamin E and A activities, respectively, are essential for the normal growth and function of the human body [1,2,3,4,5,6]. Tocopherols are synthesized by plants as α-, β-, γ-, and δ-tocopherols, which differ in the number and position of methyl groups on the chromanol ring. All four tocopherols (TOHs) can act as effective lipophilic antioxidants by donating an electron to a free radical, such as a lipid-derived peroxyl radical, thereby acting as chain-breaking antioxidants [7].

In the human diet, tocopherols are present in various types of natural food and as food additives. Alpha- and γ-tocopherols are the predominant forms of vitamin E in typical diets, coming mainly from vegetable oils [3,8]. In the intestines, all four tocopherols are incorporated into chylomicrons and secreted into the lymph and then transported to the liver. In the liver, there is a preferential uptake of α-tocopherol by the α-tocopherol transfer protein, which facilitates its incorporation into lipoproteins, which are then secreted into the systemic circulation. Gamma-, β-, and δ-tocopherols are mostly excreted into the bile or metabolized by liver enzymes into carboxyethyl hydroxychromans, which are excreted with the urine. As a result, blood levels of α-tocopherol tend to be 5 to 10-fold greater than those for γ-tocopherol; consequently, α-tocopherol is the predominant tocopherol in human and animal tissues [1]. Supplementation with other tocopherols can transiently increase their levels in blood plasma [9].

Due to its abundance in human and animal tissues, α-tocopherol has been considered as the most important inhibitor of lipid peroxidation of dietary origin in vivo; therefore, its effects on lipid peroxidation and interactions with other antioxidants have been investigated in greater detail than other tocopherols [7,10]. Due to electron donation by α-tocopherol, a resonance-stabilized phenoxyl radical (the tocopheroxyl radical) is formed. This is less reactive and does not propagate the radical chain in lipid peroxidation as readily as the lipid-derived peroxyl radicals. Then, tocopheroxyl radical can be reduced back to tocopherol by certain biological reductants, such as ascorbate (vitamin C), ubiquinol, or dihydrolipoic acid. This recycling of α-tocopherol has been proposed to account for apparent antioxidant synergism between α-tocopherol and these other antioxidants.

Furthermore, it has been also demonstrated that α-tocopherol can reduce, and thereby recycle, the semi-oxidized forms of other antioxidants, including cation radicals of two forms of vitamin A: retinol and retinoic acid [11,12,13]. Retinoid cation radicals can be formed as a result of the interaction of retinoids with hydroxyl radicals, peroxyl radicals, such as trichloromethylperoxyl radical, or the photoionization of retinoids by exposure to ultraviolet light [12,13,14,15,16].

It has been demonstrated that cation radicals of retinol and retinoic acid can damage important biomolecules, such as certain amino acids and proteins [12,13]. It has been also shown that α-tocopherol has the potential to act as an antioxidant, protecting the amino acids from oxidation by the retinol cation radical. In methanol, the bimolecular rate constant of the interaction of retinol cation radical with α-tocopherol, 2.5 × 10^9^ M^−1^·s^−1^, is two orders of magnitude greater than it is with arginine [13]. However, in oil-in-water microemulsions, the apparent bimolecular rate constants of the interaction of the retinoic acid cation radical with α-tocopherol are 26, 47, and 69-fold smaller than those for its interactions with tryptophan, lysozyme, and tyrosine, respectively [12]. This indicates that the cation radicals of different retinoids can exhibit varied reactivities toward α-tocopherol.

While retinol and retinoic acid are important, they tend to be present in human and animal tissues in smaller concentrations than another form of vitamin A: retinal [4,17,18,19,20,21]. Retinal can transiently accumulate in photoreceptive neurons in the retina, as a result of its hydrolysis from photoexcited visual pigments, before being enzymatically reduced to retinol. Exposure of the dark-adapted retina to bright light resulting in rapid bleaching by rhodopsin may cause the accumulation of retinal in millimolar concentrations. While in the retina, the retinoids are at constant risk of oxidation, they are present in environments that are rich in tocopherols [22,23,24,25,26,27,28]; therefore, it is important to determine whether radical retinal cations can be reduced by these tocopherols.

It has been established that γ-tocopherol is a better scavenger of electrophiles, such as nitrogen dioxide or peroxynitrite, than α-tocopherol [1]. This effect was ascribed to the lack of substitution at the fifth position on the chromanol ring of γ-tocopherol [1,9]. This suggests that different tocopherols can interact differently also with retinoid cation radicals.

Therefore, the aim of this study was to investigate: (i) whether γ-tocopherol and the other two tocopherols with fewer methyl group substitutions on their chromanol ring, namely β- and δ-tocopherols, can also interact with retinoid radical cations; and (ii) whether cation radicals of retinal can be reduced by tocopherols.

To achieve this, we used the pulse radiolysis of benzene solutions saturated with nitrous oxide to generate retinoid cation radicals [29,30,31], and measured the rate of decay of these species in the absence and presence of tocopherols. Based on these data, we have determined the bimolecular rate constants of scavenging for three types of retinoid cation radicals by four types of tocopherols. The results indicate that all four types of tocopherols can scavenge cation radicals of retinoids, but the bimolecular rate constants of interaction vary. The cation radical of retinal is scavenged effectively by all tocopherols with the bimolecular rate constants close to the diffusion-controlled limit. The α-, β-, and γ-tocopherols can scavenge almost as effectively as the cation radical of retinoic acid, but δ-tocopherol is about 20 times less effective. The rate constants of scavenging of retinol cation radicals by tocopherols are about 100 times smaller than for cation radicals of retinal, with δ-tocopherol again being the least effective.

## 2. Results

### 2.1. Interaction of Cation Radical of Retinal with Tocopherols Trolox and Urate

**Figure 1:**
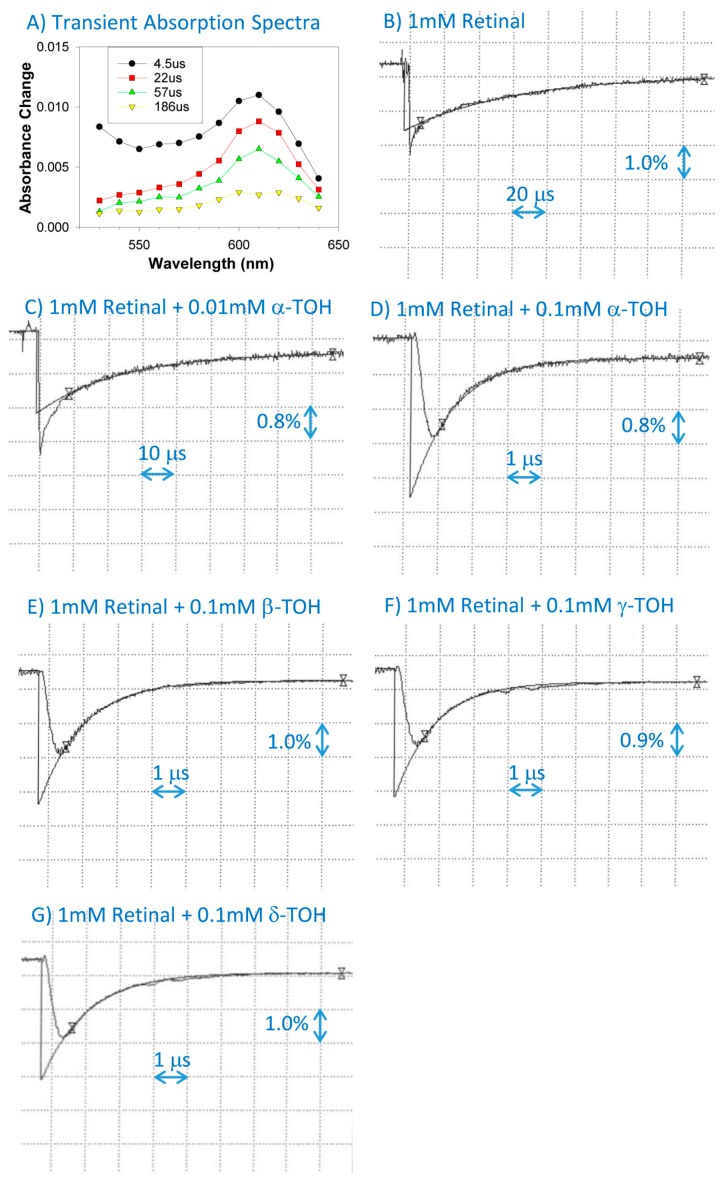
Transient absorption spectra at indicated times after the pulse radiolysis of N_2_O-saturated benzene with solubilized 1 mM of retinal (**A**), and representative kinetics of the formation and decay of retinal cation radicals monitored at 610 nm (**B**–**G**) after pulse radiolysis in the absence and presence of α-tocopherol (α-TOH), β-tocopherol (β-TOH), γ-tocopherol (γ-TOH), and δ-tocopherol (δ-TOH).

The pulse radiolysis of N_2_O-saturated benzene in the presence of 1 mM of retinal resulted in the formation of retinal cation radicals exhibiting an absorption maxima at 610 nm and decaying within 500 µs with kinetics which, after omitting the initial contribution to the decay from the retinal triplet state, could be fitted with single exponential decays (Figure 1). The cation radicals of retinal were efficiently scavenged by all four tocopherols with bimolecular rate constants of (8.0 ± 0.3), (6.7 ± 0.8), (8.1 ± 1.0), and (7.6 ± 0.5) × 10^9^ M^−1^·s^−1^ for α-, β-, γ-, and δ-tocopherol, respectively (Figure 1; Table 1).

**Figure 2:**
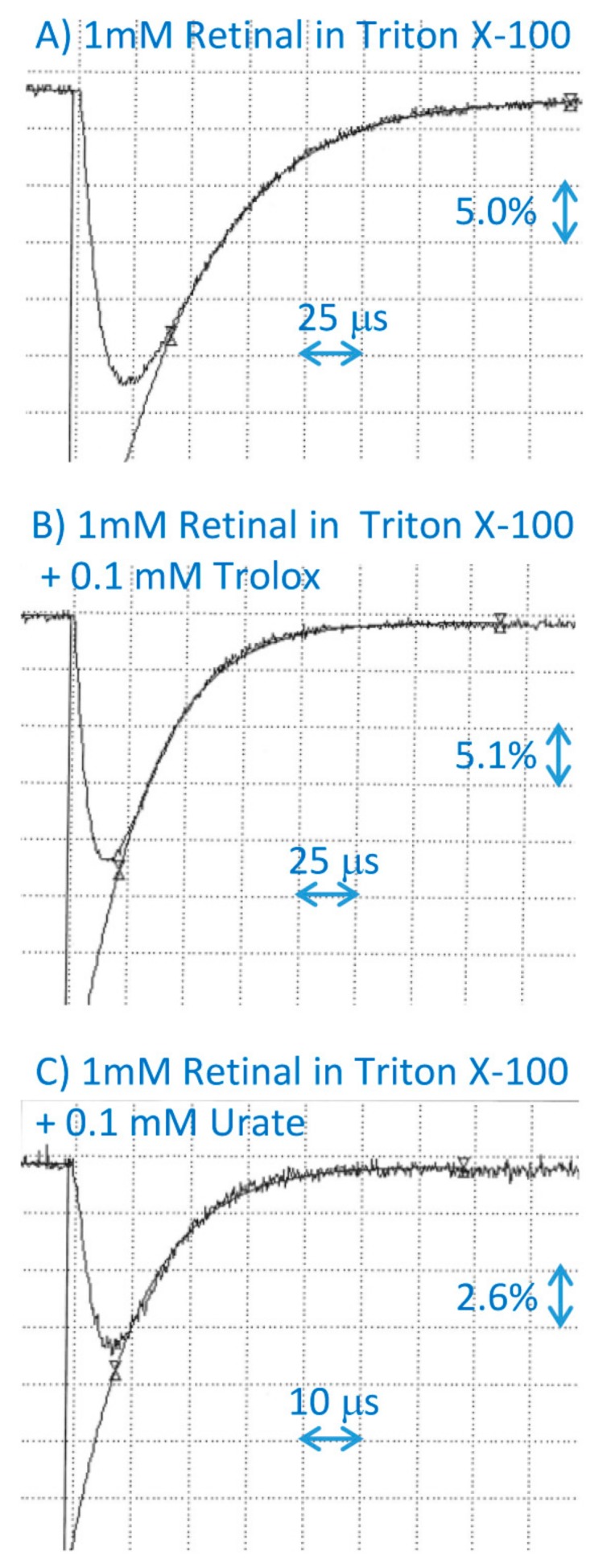
Representative kinetics of the formation and decay of retinal cation radicals monitored at 590 nm after the pulse radiolysis of aqueous solution saturated with N_2_O and containing 10 mM of phosphate, pH 7, 0.1 M KBr, 1 mM of retinal incorporated in 2% Triton X-100 micelles, in the absence (**A**) and presence of 0.1 mM of Trolox (**B**), or uric acid (**C**).

Trolox is a synthetic hydrophilic analogue of α-tocopherol, which is soluble in water; therefore, it can be used to test whether it can scavenge retinal cation radicals formed in Triton X-100 micelles as a model of the biological lipid–water interface. Trolox scavenged cation radicals of retinal in micelles with a bimolecular rate of interaction of 1.9 × 10^8^ M^−1^·s^−1^, which is 35 to 43-fold smaller than the bimolecular rates of scavenging by tocopherols when both reactants are solubilized in benzene (Figure 2; Table 1). This rate is also almost fourfold smaller than the rate of scavenging of retinal cation radicals in Triton X-100 micelles by vitamin C in the form of ascorbate [15]. It is likely that the negative charge of ascorbate anion facilitates the scavenging via electrostatic interaction with retinal radical cations. Another endogenous antioxidant that is present mostly as an anion at pH 7 is urate [32]. Therefore, we tested whether urate can also scavenge the retinal cation radicals. The urate interacted with retinal cation radicals with a bimolecular rate constant of 6.7 × 10^8^ M^−1^·s^−1^, which is similar to the rate of scavenging by ascorbate of 7.3 × 10^8^ M^−1^·s^−1^ (Figure 2; Table 1) [15].

### 2.2. Interaction of Cation Radical of Retinoic Acid with Tocopherols, Trolox and Urate

**Figure 3:**
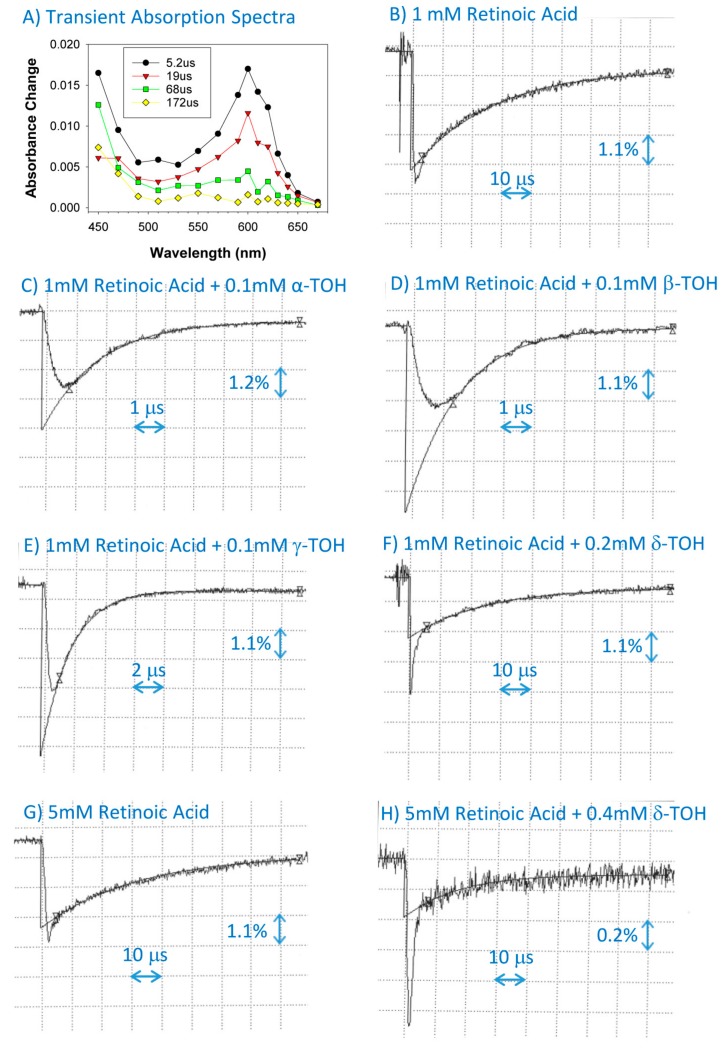
Transient absorption spectra at indicated times after pulse radiolysis of N_2_O-saturated benzene with 1 mM of solubilized retinoic acid (**A**), and representative kinetics monitored at 600 nm (**B**–**H**) of the formation and decay of retinoic acid cation radicals after pulse radiolysis in the absence and presence of α-tocopherol (α-TOH), β-tocopherol (β-TOH), γ-tocopherol (γ-TOH), and δ-tocopherol (δ-TOH).

Pulse radiolysis of N_2_O-saturated benzene in the presence of 1 mM of retinoic acid resulted in the formation of retinoic acid cation radical with an absorption maximum at 600 nm and decaying within 200 μs with a rate constant of (3.4 ± 0.4) × 10^4^ s^−1^ (Figure 3; Table 1). The rate of decay was substantially increased in the presence of 0.1 mM of α-, β-, and γ-tocopherols with bimolecular rate constants of (5.56 ± 0.24) × 10^9^, (5.52 ± 0.24) × 10^9^, and (4.72 ± 0.22) × 10^9^ M^−1^·s^−1^, respectively. There was no increase in the rate of decay of the retinoic acid cation radical in the presence of 0.1 and 0.2 mM of δ-tocopherol. Increasing concentrations of δ-tocopherol to 0.4 and 1.0 mM in solutions of 5 mM of retinoid acid resulted in small increases in the rate of decay, giving the bimolecular rate constant of interaction of (2.8 ± 2.2) × 10^7^ M^−1^·s^−1^. Clearly, δ-tocopherol is two orders of magnitude less efficient than other tocopherols regarding scavenging semi-oxidized retinoic acid.

**Figure 4:**
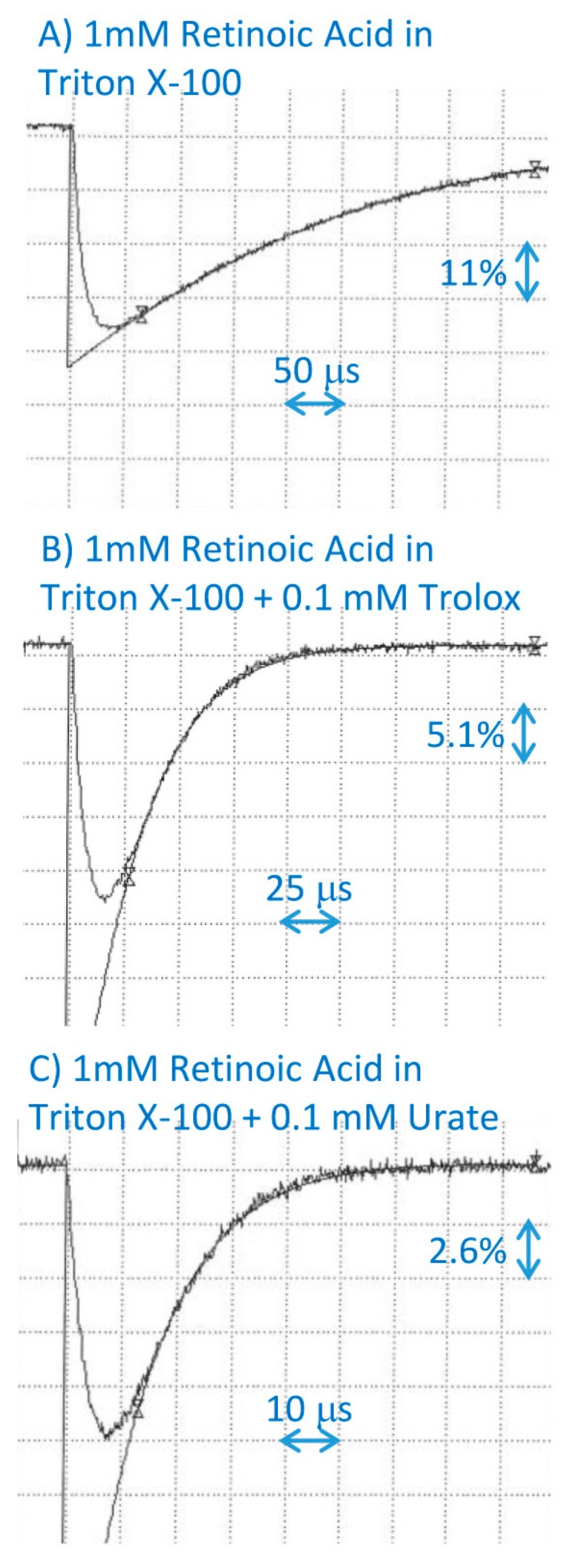
Representative kinetics of the formation and decay of retinoic acid cation radicals monitored at 590 nm after pulse radiolysis of aqueous solution saturated with N_2_O and containing 10 mM of phosphate, pH 7, 0.1 M of KBr, and 1 mM of retinoic acid incorporated in 2% Triton X-100 micelles, in the absence (**A**) and presence of 0.1 mM of Trolox (**B**) or uric acid (**C**).

Trolox scavenged the cation radicals of retinoic acid with the bimolecular rate constant of 3.6 × 10^8^ M^−1^·s^−1^, which is 13 to 16 times less efficient than α-, β-, and γ-tocopherols, but 13 times faster than δ-tocopherol (Figure 4; Table 1). The urate scavenged retinoic acid cation radicals with the bimolecular rate constant of 7.8 × 10^8^ M^−1^·s^−1^, which is 2.2-fold more efficient than the interaction with Trolox (Figure 4; Table 1), and slightly more efficient than with ascorbate, for which the rate of scavenging is 6.5 × 10^8^ M^−1^·s^−1^ [15].

### 2.3. Interaction of Tocopherols with Cation Radical of Retinol

**Figure 5:**
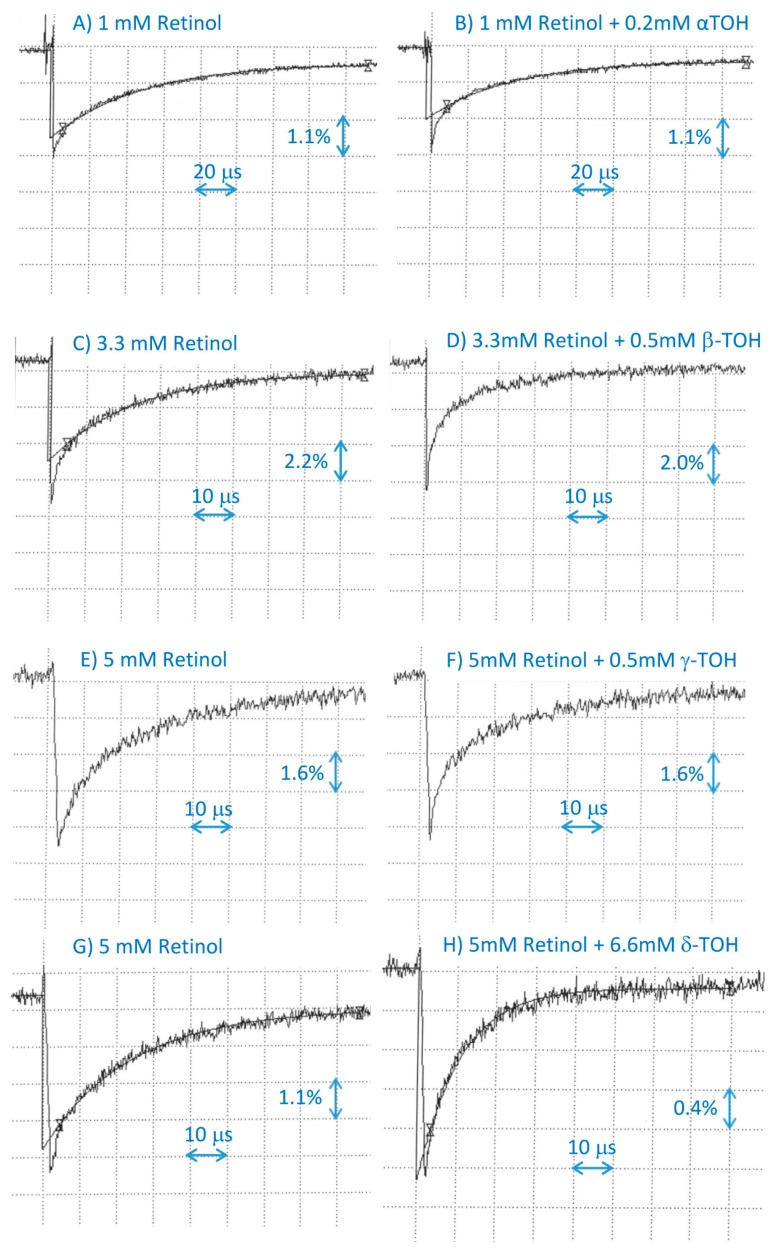
Representative kinetics of the formation and decay of retinol cation radicals monitored at 610 nm after pulse radiolysis of N_2_O-saturated benzene with solubilized retinol at indicated concentrations in the absence (**A**,**C**,**E**,**G**)and presence of α-tocopherol (α-TOH, **B**), β-tocopherol (β-TOH, **D**), γ-tocopherol (γ-TOH, **F**), and δ-tocopherol (δ-TOH, **H**).

Pulse radiolysis of N_2_O-saturated benzene in the presence of 1 mM of retinol resulted in the formation of retinol cation radicals exhibiting an absorption maxima at 610 nm and decaying within 200 µs with kinetics which, after omitting the initial contribution to the decay from the retinol triplet state, could be fitted with a single exponential decay (Figure 5). The rate of decay of the retinol cation radical was considerably increased in the presence of sub-millimolar concentrations of α-tocopherol, allowing the determination of the corresponding bimolecular rate constant of interaction in benzene as 8.0 × 10^7^ M^−1^·s^−1^ (Figure 5; Table 1).

Beta-tocopherol at a concentration of 0.1 mM exerted only a small effect on the rate of decay of the retinol cation radicals; therefore, to explore whether they interact, the concentrations of the retinol and β-tocopherol were increased to 3.3 mM and up to 0.5 mM, respectively (Figure 5; Table 1). The rate of scavenging of the retinol cation radical by β-tocopherol was (1.6 ± 0.9) × 10^8^ M^−1^·s^−1^.

Also, the effect of 0.1 mM of γ-tocopherol on the rate of decay of retinol cation radicals was rather small, so the concentrations of retinol and γ-tocopherol were increased to 5 mM and 0.5 mM, respectively, and the rate of scavenging of the retinol cation radical by γ-tocopherol was determined as 1.0 × 10^7^ M^−1^·s^−1^ (Figure 5; Table 1).

Delta-tocopherol at a concentration of 0.2 mM did not increase the rate of decay of retinol cation radicals; therefore, to explore whether they interact, the concentration of retinol was increased to 5 mM, and δ-tocopherol was increased to 1 mM and higher (Figure 5; Table 1). These high concentrations of δ-tocopherol resulted in an effective competition with retinol for free radicals formed due to the radiolysis of benzene, thereby decreasing the yield of retinol cation radicals. Still, there was a linear relationship between the apparent rate of decay of retinol cation radicals and the concentration of δ-tocopherol. The rate of scavenging of retinol cation radicals by δ-tocopherol was (7.7 ± 1.2) × 10^6^ M^−1^·s^−1^.

**Figure 6:**
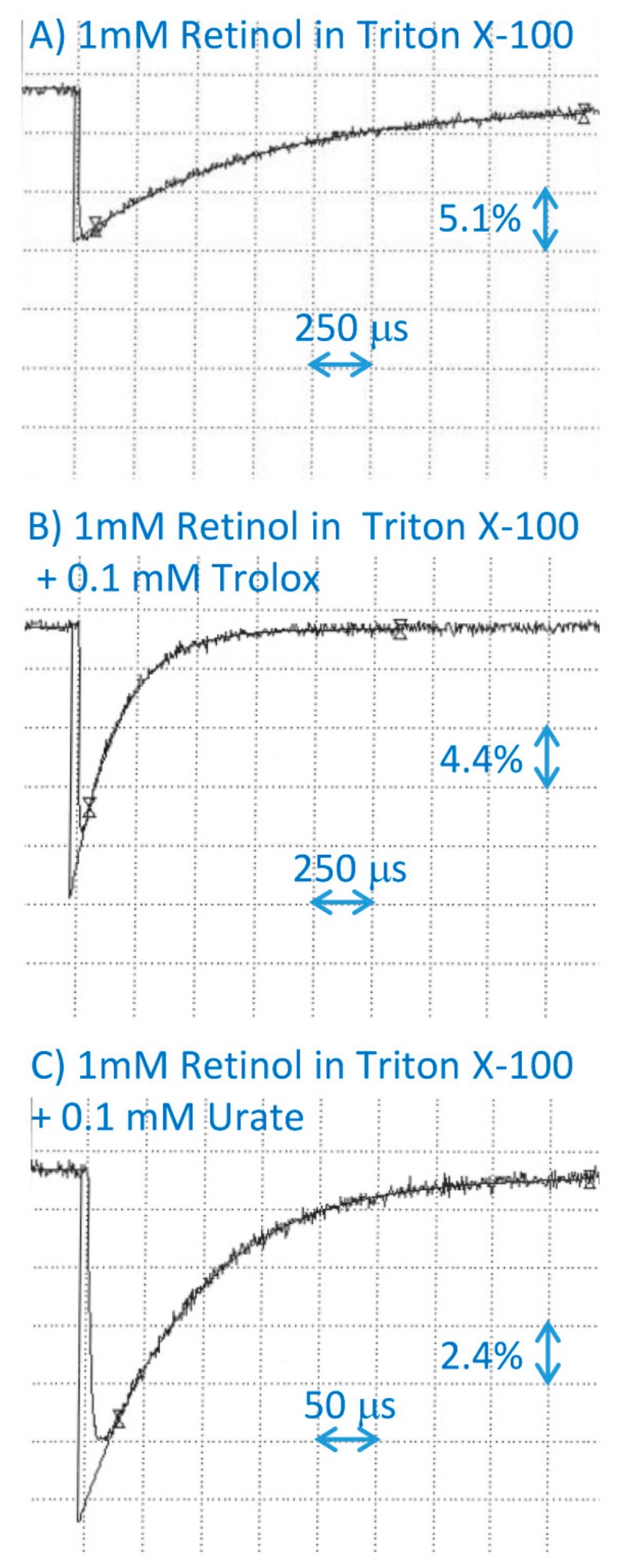
Representative kinetics of the formation and decay of retinol cation radicals monitored at 590 nm after pulse radiolysis of aqueous solution saturated with N_2_O and containing 10 mM of phosphate, pH 7, 0.1 M of KBr, and 1 mM of retinol incorporated in 2% Triton X-100 micelles, in the absence (**A**) and presence of 0.1 mM of Trolox (**B**) or uric acid (**C**).

El-Agamey et al. determined that the rates of scavenging of retinol cation radicals by α-tocopherol and Trolox were 2.5 and 2.3 × 10^9^ M^−1^·s^−1^, respectively, showing that they are similar when both reactants are solubilized in methanol [13]. Our results show that Trolox scavenged cation radicals of retinol in micelles with the bimolecular rate constant of 4.6 × 10^7^ M^−1^·s^−1^ (Figure 6; Table 1), which is close to the bimolecular rate constant of α-tocopherol interaction with the retinol cation radicals in benzene (Figure 4; Table 1).

Urate scavenged retinol cation radicals in micelles with the bimolecular rate constant of 1.1 × 10^8^ M^−1^·s^−1^ (Figure 6; Table 1), which is the same within the experimental uncertainty as the rate of scavenging by ascorbate [15].

## 3. Discussion

In summary, all four types of tocopherol can scavenge cation radicals of retinoids, with the highest bimolecular rate constants for the scavenging of retinal cation radicals ranging from 7 to 8 × 10^9^ M^−1^·s^−1^, which are close to the diffusion-controlled limits. Cation radicals of retinoic acid are scavenged effectively by α-, β-, and γ-tocopherols with bimolecular rate constants from 5 to 6 × 10^9^ M^−1^·s^−1^, whereas δ-tocopherol scavenged the retinoic acid cation radicals with the rate constant of only ~3 × 10^7^ M^−1^·s^−1^, thus being about 200 times less effective than the other three tocopherols. Tocopherols were two to three orders of magnitude less efficient at scavenging radical cations of retinol than retinal and retinoic acid, with δ-tocopherol being the least active.

Our data demonstrate that α-tocopherol scavenges cation radicals of retinoic acid in benzene with a bimolecular rate constant that is over three orders of magnitude higher than the apparent rate constant of scavenging of retinoic acid cation radicals by α-tocopherol that was observed in the oil-in-water microemulsion by Li et al., which was only (3.9 ± 0.3) × 10^6^ M^−1^·s^−1^ [12]. This supports the explanation provided by Li et al. that the spatial arrangement of α-tocopherol versus the retinoid radical cation can play a role in their interaction, with the relatively polar hydroxyl group of the α-tocopherol and charged part of the retinoid radical cation being both close to the water–oil interface, which clearly hinders their interaction in comparison to their free diffusion in a non-polar solvent.

On the other hand, our data demonstrate that the bimolecular rate constant of scavenging of retinol cation radical by α-tocopherol in benzene is 31-fold smaller than the rate constant of scavenging measured in methanol [13], suggesting that polar solvents facilitate the interaction better than non-polar solvents.

The scavenging of retinoid cation radicals by tocopherols can have important implications for physiology and pathophysiology where it can be the basis for the co-operation of vitamins A and E. It has been demonstrated in several studies that retinoids are more effective than vitamin E at inhibiting free radical-induced lipid peroxidation and the formation of malondialdehyde in the outer rod segments from bovine retina and rat brain mitochondria [33,34]. However, during that process, retinoids undergo degradation [35,36,37]. It has been shown that retinol degradation can be inhibited by α-tocopherol during free radical-induced lipid peroxidation in liposomes and bovine retinal membranes. This protection may be, at least in part, attributed to the regeneration of the retinoid from the semi-oxidized state. These findings together with our data indicate a potential mechanism by which retinoids and tocopherols can co-operate: they both can scavenge initiating radicals and lipid-derived radicals; the cation radicals of retinoids can be scavenged by tocopherols, resulting in a less reactive radical. This may be of importance for the skin, where retinoids are often used as topical or systemic treatments [38,39,40,41], but are prone to photodegradation and the generation of products with pro-oxidant activities [42,43]. Therefore, ensuring that tocopherols are in the proximity of retinoids may prevent retinoid degradation and its deleterious consequences for the skin.

Our findings showing that tocopherols can scavenge semi-oxidized retinal are of particular importance for the retina, where high levels of retinal can accumulate in photoreceptor neurons upon exposure of the dark-adapted retina to bright light [4,17,18,19,20,21]. Upon the absorption of ultraviolet or blue light, retinal can photosensitize the formation of singlet oxygen and free radicals, which can initiate lipid peroxidation as well as the oxidation of retinal. The parts of photoreceptor neurons where retinal accumulates are rich in polyunsaturated lipids and weakly chelated iron ions, making it a vulnerable environment for the propagation of lipid peroxidation and the degradation of retinal. We have shown that after photooxidation, degradation products of retinal retain its photosensitizing properties and becomes more cytotoxic than retinal in the dark [44]. Therefore, it is important to prevent retinal from oxidizing. The results presented in this manuscript suggest that all the tocopherols, Trolox and urate can scavenge retinal cation radicals, and thereby provide a potential recycling mechanism where the semi-oxidized retinoid is converted back to the original compound.

The increased accumulation of retinal in the retina is thought to contribute to pathological changes in inherited blinding diseases such as Stargardt’s disease (STGD1) and age-related macular degeneration (AMD) [18,19,20,21]. STGD1 is the most common inherited macular dystrophy in children and young adults [45,46,47]. It is caused by pathogenic variants in the gene coding ATP binding cassette transporter type A4 (ABCA4). ABCA4 is highly expressed in the rims of photoreceptor discs and at much lower levels in the phagolysosomes of the retinal pigment epithelium (RPE), where it facilitates the clearance of retinal by facilitating its reduction to retinol, and by its removal from phagocytosed discs, enabling its binding to the chaperone protein, respectively [48,49]. Mutations of the *ABCA4* gene resulting in the loss of function of its product lead to the delayed clearance of retinal and the accumulation of bisretinoids, which could contribute to the phototoxicity of the retina.

AMD is the leading cause of vision loss in elderly people [50,51]. Initially, it is characterized by pigmentary changes and the formation of deposits between the RPE and Bruch’s membrane, which separates the outer retina from its choroidal blood supply. Then, it progresses to the atrophic form where RPE cells and photoreceptors die, forming areas in the retina that are no longer responsive to light, or to the neovascular form, where the choroidal blood vessels grow into the retina, forming exudates and causing fluid accumulation. While the advanced age is the greatest risk factor for this disease, other risk factors include smoking, an increased lifetime exposure to sunlight, a low dietary intake of antioxidants, and certain gene variants [52]. The accumulation of retinal has been considered a primary step in creating increased oxidative stress, leading to the formation of oxidation products, which can activate damaging complement cascades, as well as pro-inflammatory and pro-angiogenic pathways [19,20,52,53]. Epidemiological evidence indicates that there is an association between an increased intake of vitamin A and the early stage of AMD in people with AMD-associated polymorphisms in complement factor H, which appears to exacerbate damage under increased oxidative stress [54,55,56].

Several lines of evidence indicate that vitamin E does play an important role in the retina and can protect it from oxidation [22,28,57,58,59,60,61]. Some, but not all, epidemiological studies indicate that an increased dietary intake of vitamin E is protective against the development of AMD [62,63,64], whereas vitamin E supplements in combination with vitamin C, carotenoids, and zinc do not prevent AMD development, but do slow down its progression from a moderate to advanced form by 25% [65,66].

Both STGD1 and AMD are associated with increased oxidative stress and the damaging effects of vitamin A or its derivatives. The formation of retinoid cation radicals is a potential pathway contributing to these damaging effects, while the ability of tocopherols and vitamin C to scavenge these radicals may explain their protective effects that are seen in epidemiological studies and clinical trials.

In conclusion, our results provide a potential mechanism by which tocopherols can diminish the oxidative damage to the skin and retina by scavenging retinoid cation radicals, and thereby protecting from skin photosensitivity and the development and/or progression of changes in Stargardt’s disease and AMD.

## 4. Materials and Methods

### 4.1. Chemicals

All retinoids—all-*trans*-retinoic acid, all-*trans*-retinal, and all-*trans*-retinol—Trolox, uric acid, and Triton X-100 were supplied by Sigma-Aldrich; α-, β-, γ-, and δ-tocopherols were obtained from Merck KGaA, Darmstadt, Germany. Analar-grade Na_2_HPO_4_ and NaH_2_PO_4_ were from BDH (VWR International Ltd., Dorset, UK). Water was redistilled from alkaline permanganate.

### 4.2. Pulse Radiolysis

Pulse radiolysis is a technique for generating free radicals and excited states, and for analyzing their interactions with other molecules via monitoring changes in the absorption of light [67]. The pulse radiolysis experiments were carried out with a 9–12 MeV Vickers linear accelerator, as previously described [15,68], using a single monitoring wavelength for each 20 to 500-ns pulse of electron beam. Solutions were studied using quartz flow-through cells with an optical path length of 2.5 cm. The detection system consisted of a high-pressure compact arc xenon lamp, a pulsing unit, a high-radiance Kratos monochromator, and quartz optics. Optical transmissions at various wavelengths selected with the monochromator, which had bandwidths between 10–40 nm, were observed as a function of time before and after the electron pulse using photoelectric detection. The output of the photomultiplier (EMI 9558Q) was displayed on a Tektronix TDS 380 digitizing oscilloscope. Data processing was performed on a Dan PC using software developed in-house. Radiation doses were measured using the thiocyanate dosimeter, by which the absorption of (SCN)_2_^●−^ formed by pulsing an air-saturated aqueous solution of 10^−2^ M KSCN was measured by taking an absorption coefficient of (SCN)_2_^●−^ of 7.1 × 10^3^ M^−1^·cm^-1^ and a G value of 0.30 μM·Gy^−1^ [69].

### 4.3. Generation of Retinoid Cation Radicals

To generate retinoid cation radicals in benzene, retinoids were solubilized in N_2_O-saturated benzene. Following the pulse radiolysis of the solvent, retinoid radical cations were formed by a positive charge transfer from benzene cations, while N_2_O prevented the formation of radical anion by electron capture according to the sequence [68,70]:

C_6_H_6_ → C_6_H_6_^●+^ + e^−^

e^−^ + N_2_O → N_2_ + O^●−^

O^●−^ + C_6_H_6_ → C_6_H_6_O^●−^

C_6_H_6_O^●−^ + N_2_O → N_2_ + C_6_H_6_O_2_^●−^

C_6_H_6_O_2_^●−^ + C_6_H_6_ → C_6_H_5_OH + C_6_H_6_O

C_6_H_6_^●+^ + Retinoid → C_6_H_6_ + Retinoid^●+^

To generate retinoid cation radicals in Triton X-100 micelles, 1-mM retinoids were incorporated in 2% Triton X-100 micelles solubilized in 10 mM of phosphate buffer, pH 7.0, as described previously [15]. Retinoid cation radicals were generated by the pulse radiolysis of N_2_0 saturated water in the presence of 0.1 M of potassium bromide according to the sequence [15]:

H_2_O → e_aq_^−^ + OH^●^

e_aq_^−^ + N_2_O + H_2_O → N_2_+ OH^●^ + OH^−^

OH^●^ + Br^−^ → Br^●^ + OH^−^

Br^●^ + Br^−^ → Br_2_^●−^

Br_2_^●−^ + Retinoid → 2Br^−^ + Retinoid^●+^

### 4.4. Interaction of Retinoid Cation Radicals with Antioxidants

In order to study the interaction of retinoid cation radicals with lipophilic antioxidants, both retinoids and tocopherols were solubilized in benzene and saturated with nitrous oxide. Initially, retinoids were studied at concentrations of 1 mM, while tocopherols were studied at concentrations of 0.1 or 0.2 mM. A one-millimolar concentration of retinoids is sufficient to generate easily measurable retinoid cation radicals with high signal-to-noise ratios. Concentrations of tocopherols need to be several fold smaller than the retinoid concentration to ensure that the formation of retinoid cation radicals predominates over the tocopheroxyl radicals. When no quenching of retinoid cation radicals was observed at these concentrations, the concentration of retinoids was increased, allowing an increased concentration of tocopherol to be used, whilst still allowing the formation of retinoid cation radicals from the solvent cations to predominate.

To study the interaction of retinoid cation radicals with hydrophilic antioxidants, 1-mM retinoids were incorporated into Triton X-100 micelles, whereas Trolox or uric acid were solubilized directly in phosphate buffer.

The decays of retinoid cation radicals were measured in the absence and presence of antioxidants, and fitted with exponential decay functions to obtain the rate constants of their first-order decays. The bimolecular rate constants of quenching retinoid cation radicals by tocopherols were calculated from the slope of the linear fit of the rate of decay versus antioxidant concentration.

## Figures and Tables

**Table 1:** Bimolecular rates of scavenging of retinoid cation radicals by tocopherols, trolox and urate. To determine the bimolecular rate constants of interactions between tocopherols and retinoid cation radicals, both reactants were solubilized in benzene. To determine the bimolecular rate constants of interactions between retinoid cation radicals and trolox or urate, the retinoids were incorporated into Triton X-100 micelles, whereas hydrophilic antioxidants were solubilized directly in 10 mM of phosphate buffer pH 7.0.

	Bimolecular Rates of Scavenging of Retinoid Radical Cations (10^8^ M^−1^·s^−1^)		
	Retinal Cation Radical	Retinoic Acid Cation Radical	Retinol Cation Radical
α-Tocopherol	80.2 ± 3.2	55.6 ± 2.4	0.80 ± 0.45
β-Tocopherol	67.3 ± 8.3	55.2 ± 2.4	1.6 ± 0.9
γ-Tocopherol	81.3 ± 10.2	47.2 ± 2.2	0.10 ± 0.06
δ-Tocopherol	76.0 ± 4.5	0.28 ± 0.22	0.08 ± 0.01
Trolox	1.90 ± 0.85	3.57 ± 0.04	0.46 ± 0.03
Urate	6.66 ± 0.02	7.81 ± 0.01	1.10 ± 0.10
